# Transcription factor KROX20 marks epithelial stem cell ancestors for hair follicle formation

**DOI:** 10.1172/JCI180160

**Published:** 2024-10-03

**Authors:** Elnaz Ghotbi, Edem Tchegnon, Zhiguo Chen, Stephen Li, Tracey Shipman, Yong Wang, Jenny Raman, Yumeng Zhang, Renee M. McKay, Chung-Ping Liao, Lu Q. Le

**Affiliations:** 1 Department of Dermatology,; 2 Genetics, Development and Disease Graduate Program, and; 3 Medical Scientist Training Program, University of Texas Southwestern Medical Center, Dallas, Texas, USA.; 4 Graduate Institute of Medical Sciences and; 5 International PhD Program in Cell Therapy and Regenerative Medicine, College of Medicine, Taipei Medical University, Taipei, Taiwan.; 6 Hamon Center for Regenerative Science and Medicine and; 7 Simmons Comprehensive Cancer Center, University of Texas Southwestern Medical Center, Dallas, Texas, USA.; 8 Department of Dermatology, University of Virginia School of Medicine, Charlottesville, Virginia, USA.

**Keywords:** Development, Stem cells, Mouse stem cells

## Abstract

Epidermal stem cells control homeostasis and regeneration of skin and hair. In the hair follicle (HF) bulge of mammals, populations of slow-cycling stem cells regenerate the HF during cyclical rounds of anagen (growth), catagen (regression), and telogen (quiescence). Multipotent epidermal cells are also present in the HF above the bulge area, contributing to the formation and maintenance of sebaceous gland and upper and middle portions of the HF. Here, we report that the transcription factor KROX20 is enriched in an epidermal stem cell population located in the upper/middle HF. Expression analyses and lineage tracing using inducible *Krox20-CreERT* showed that *Krox20*-lineage cells migrate out of this HF region and contribute to the formation of the bulge in the HF, serving as ancestors of bulge stem cells. In vivo depletion of these cells arrests HF morphogenesis. This study identifies a marker for an epidermal stem cell population that is indispensable for hair homeostasis.

## Abstract 2

This study identifies a novel epidermal stem cell population required for hair formation and regeneration, a finding that could lead to possible treatment for balding.

## Graphical abstract

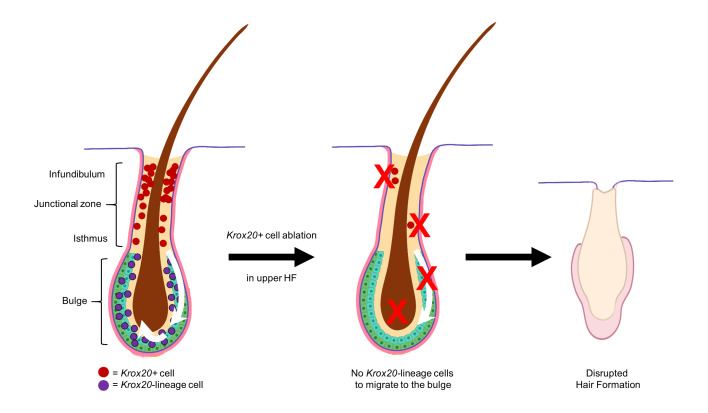

## Introduction

Adult stem cells play a critical role in normal tissue homeostasis and regeneration (1). Correction of mutant gene expression in epidermal stem cells can reverse the disease state of the genetic blistering disorder epidermolysis bullosa (2), highlighting the importance of stem cell plasticity and its role in the treatment of genetic diseases. The precise identification of stem cell locations or niches is essential to elucidate how these cells contribute to tissue health and function (3, 4). In the skin, the hair follicle (HF) bulge is occupied by slow-cycling cells marked by CD34 and K15 (5–7). Bulge stem cells were originally thought to be the universal stem cells for the HF and interfollicular epithelium (IFE), because they were identified as the only label-retaining cell population in the epidermis (5). However, subsequent studies showed that bulge stem cells only give rise to the HF but not the IFE during normal homeostasis (8), and immediate stem/progenitor cells for IFE are generally believed to reside in the basal layer of epidermis.

The HF structures from the bulge to the IFE are composed of the isthmus, junctional zone, and infundibulum. Including the bulge, these HF compartments are collectively defined as the permanent portion of the HF, as they do not change throughout the hair cycle (9). Previously reported markers for permanent HF niches include LGR6 (isthmus) (10), LRIG1 (junctional zone) (11), and SCA1 (infundibulum) (12). During adult homeostasis, *Lrig1*-positive cells give rise to the infundibulum and sebaceous glands (13), and *Sca1*-positive cells contribute to the maintenance of IFE and infundibulum (12). Nevertheless, to the best of our knowledge, there is no documented stem cell population within the permanent regions of the upper and middle HF that contributes to the formation of the bulge during normal adult homeostasis.

In this study, we discovered that the transcription factor *Krox20* (*EGR2*) is enriched in an epidermal stem cell population located in the upper and middle HF. Using genetic labeling for cell lineage tracing, we found that *Krox20*-lineage cells contribute to the formation of the bulge and hair shaft, and that their depletion in vivo disrupts hair homeostasis.

## Results

### Krox20 marks an epidermal stem cell population in the HF.

**Figure 1:**
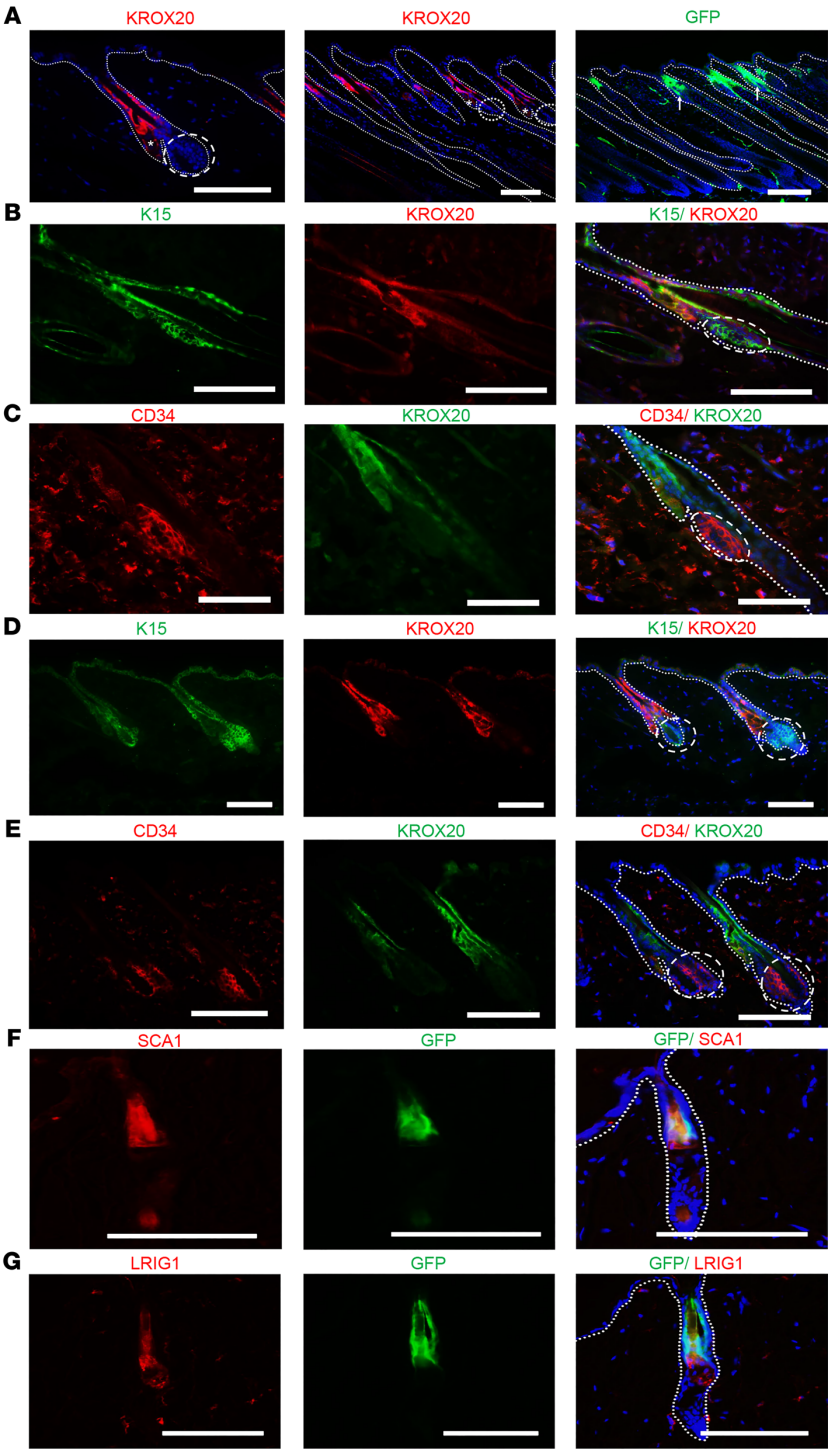
Comparative analysis of *Krox20* expression in telogen and anagen HFs in relation to other stem cell niches within the HF. (**A**) Immunofluorescence analysis depicting *Krox20* expression with KROX20 antibody in telogen (left) and anagen (middle) and by GFP immunofluorescence in anagen HFs of *Krox20-GFP* mice (right). (**B**–**E**) Colocalization analysis demonstrating the absence of colocalization between *Krox20* expression and bulge stem cell markers, K15 and CD34, in anagen (**B** and **C**) and telogen (**D** and **E**) HFs. (**F** and **G**) Colocalization analysis of GFP expression with upper stem cell niche markers such as SCA1 (**F**) and LRIG1 (**G**) in telogen HFs of *Krox20-GFP* mice. All dashed circles and ovals represent the bulge area. Arrows point to autofluorescence from hair shafts. Asterisks represent the sebaceous glands. *n* ≥ 3. Scale bars: 100 mm.

We recently reported that the transcription factor *Krox20* is a lineage marker for hair shaft structural cells during HF morphogenesis (14). To gain further insight into the developmental origin of *Krox20*-positive cells and explore their fate within the skin, we first set out to determine the exact expression pattern of *Krox20* during late embryonic and postnatal development. Subsequently, we aimed to perform lineage tracing of *Krox20*-positive cells to track their lineage and observe their behavior. We used a *Krox20-GFP* knockin mouse line (15) to monitor live *Krox20* expression with a GFP reporter. Since this line is a “knockin” and not a transgenic, GFP expression faithfully recapitulates endogenous *Krox20* expression, as validated through immunohistochemistry using a KROX20 antibody (Supplemental Figure 1A; supplemental material available online with this article; https://doi.org/10.1172/JCI180160DS1). We observed initial expression of *Krox20* in the infundibulum of mouse embryonic whisker HF at E14.5 (Supplemental Figure 1B). Subsequently, *Krox20* expression was observed in all skin HFs, persisting throughout postnatal development (Supplemental Figure 1B). Histological analysis of mouse skin at various postnatal ages revealed specific regions exhibiting *Krox20* expression. The expression was found to be prominent in the infundibulum and upper HF, extending to the sebaceous glands and the middle portions of the telogen and anagen HFs (Figure 1 and Supplemental Figure 1C). Notably, the expression of *Krox20* was enriched in the upper HF and extended across various layers of HF but was restricted to the innermost layer of the outer root sheath in the middle HF (Figure 1, A–E, and Supplemental Figure 1C) (16).

In order to characterize the relative location of *Krox20-*positive cells with respect to previously identified stem cell niches within the HF, we performed immunostaining of the skin from *Krox20-GFP* mice with bulge stem cell markers (K15 and CD34), an infundibulum marker (SCA1), and a junctional zone marker (LRIG1). We observed no colocalization between the KROX20 protein and the bulge stem cell markers, K15 or CD34, in either anagen or telogen HFs (Figure 1, B–E). This indicates that the cell population marked by KROX20 is distinct from the bulge stem cells. Instead, KROX20 exhibited a degree of overlapping expression with LRIG1- and SCA1-expressing cells (Figure 1, F and G). Upon examination of the expression domain of telogen HF, it became apparent that *Krox20* expression was enriched in the upper and middle portions of the telogen HF, including the infundibulum and junctional zone, as indicated by the overlap with SCA1 and LRIG1 markers (Figure 1, F and G). Additionally, based on the expression domain of KROX20 itself, it appeared to extend to the isthmus and sebaceous glands (Figure 1).

### Krox20-lineage cells give rise to the HF bulge.

**Figure 2:**
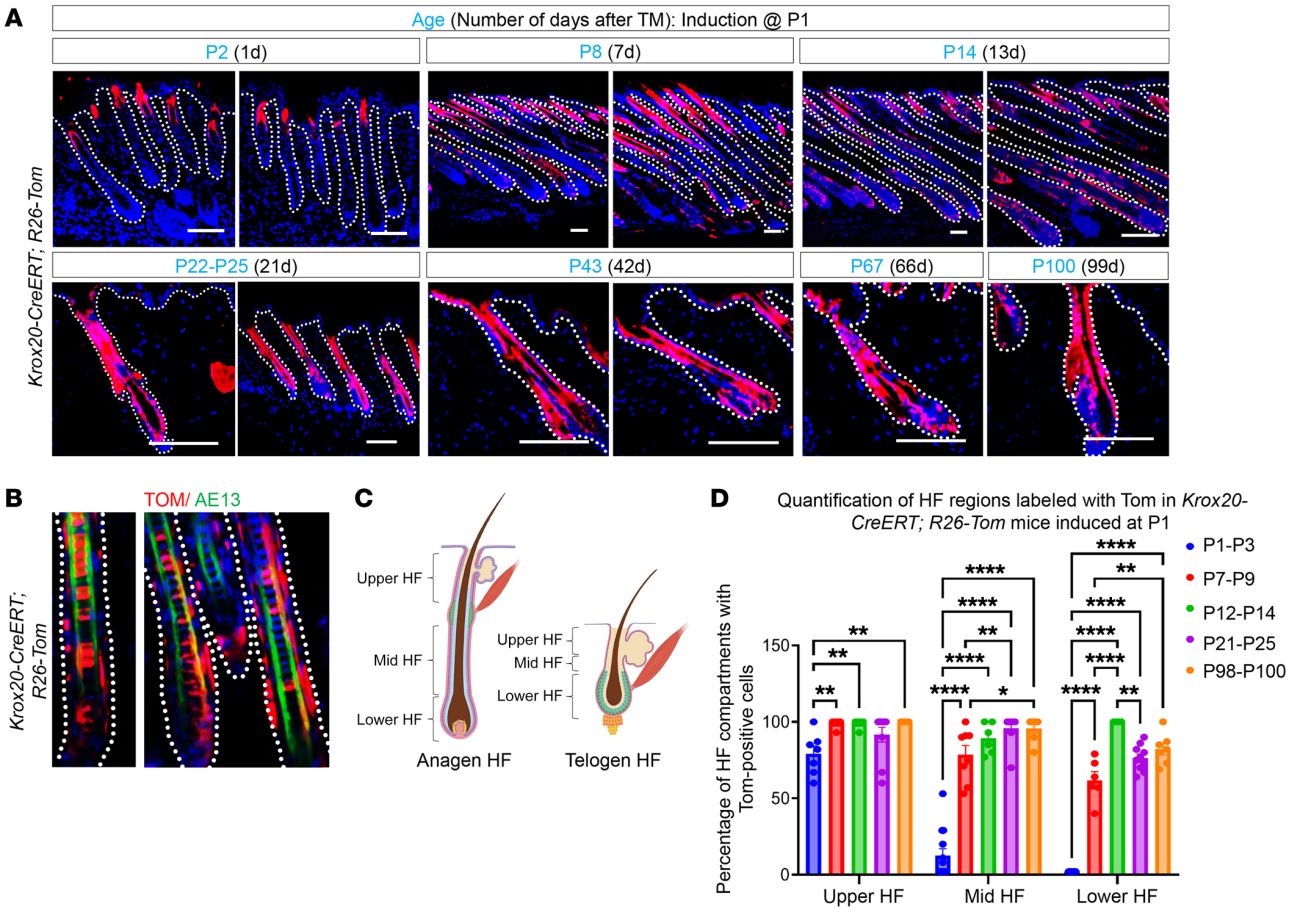
Lineage tracing with an inducible *Krox20-CreERT* confirms the contribution of *Krox20*-lineage cells to the HF bulge. (**A**) *Krox20-CreERT; R26-tdTomato* mice were induced with tamoxifen at P1. Lineage tracing shows that *Krox20*-lineage cells are initially restricted to the upper and middle HF but, over time, they expand downward, contributing to the formation of the bulge and hair shaft. (**B**) Colocalization analysis of *Krox20*-lineage cells with hair shaft marker AE13. (**C**) Diagram of an anagen HF and a telogen HF. (**D**) Quantification of HF regions labeled with *tdTomato* at various time points in *Krox20-CreERT; R26-tdTomato* mice when induced at P1. *n* ≥ 3. Scale bars: 100 μm. Tom, *tdTomato*. Statistical significance was determined for **D** by 2-way ANOVA; statistics represent mean ± SEM, *P < 0.05, **P < 0.01, ****P < 0.0001. Non-significant values were not plotted on the graphs due to space constraints.

To determine the fate of these *Krox20*-positive cells, we performed lineage tracing. As an inducible Cre driver is the gold standard tool for lineage tracing, we first generated an inducible *Krox20-CreERT* knockin mouse line. Using CRISPR/Cas9, *CreERT* was inserted after the last coding sequence of *Krox20*, following a P2A linker. The P2A linker is a short peptide sequence of about 18–25 amino acids that causes ribosome skipping (17, 18), resulting in the separate translation of the upstream protein and the downstream protein, in this case, KROX20 and CreERT. This design ensures that *Krox20* expression and function are preserved in mouse lines containing *Krox20-CreERT* (Supplemental Figure 2A). We crossed this line with *R26-tdTomato* reporter mice to generate *Krox20-CreERT; R26-tdTomato* mice. Notably, in the absence of tamoxifen treatment, minimal *tdTomato* signal was observed, demonstrating tight regulation with minimal leakiness (Supplemental Figure 2, B–D). Mice were then treated with 1 dose of tamoxifen perinatally, and their skin was subsequently analyzed at various time points, starting at 1 day after induction and continuing throughout adulthood (Figure 2A). We observed that *Krox20*-lineage cells in the *Krox20-CreERT; R26-tdTomato* lineage tracing were initially enriched in the upper and middle HF perinatally. However, over time, these cells gradually gave rise to the bulge region and were also detected along the IFE (Figure 2A). Costaining of *tdTomato* and AE13, a marker of hair shaft, indicated that *Krox20-*lineage cells contributed to the formation of the hair shaft as early as P12–P14 (Figure 2B). Quantification of HF regions labeled with *tdTomato* at various time points in anagen and telogen HFs (Figure 2C) of *Krox20-CreERT; R26-tdTomato* mice when induced at P1 is shown in Figure 2D.

**Figure 3:**
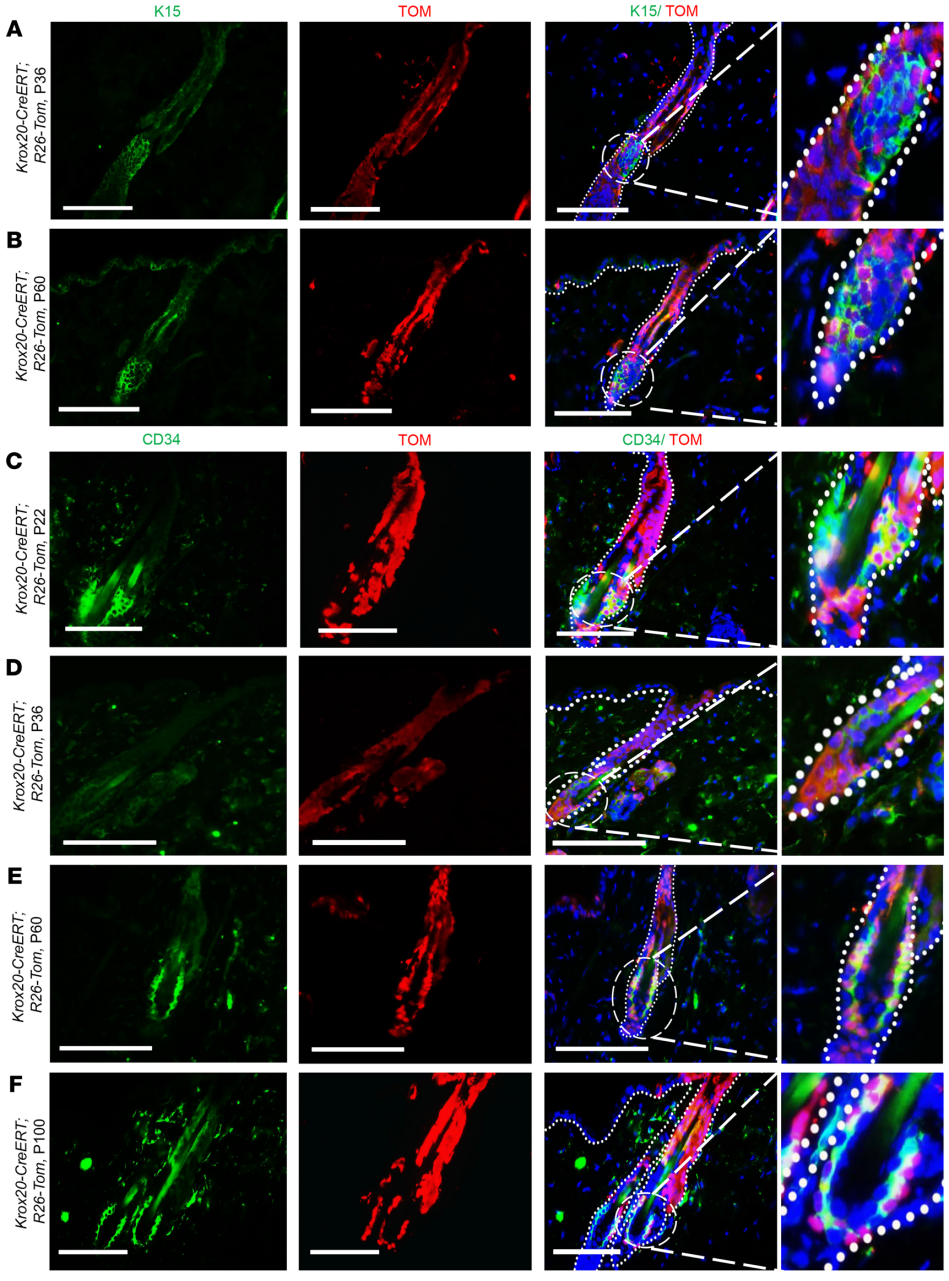
*Krox20* marks an epidermal stem cell niche in the HF excluding the bulge region. Colocalization analysis showing *Krox20*-lineage cells in relation to bulge stem cell markers K15 (**A** and **B**) and CD34 (**C**–**F**) within anagen (P36, **A** and **D**) and telogen (P60, **B**; P22, **C**; P60, **E**; and P100, **F**) HFs, induced at P1. Dashed circles represent the bulge area, which is shown at higher magnification in the panels at the end of each row (×400 for **A**–**C**, **E**, and **F**, and ×200 for **D**). *n* ≥ 3. Scale bars: 100 μm. TOM, *tdTomato*.

The presence of *Krox20*-lineage cells along the HF, especially their detection in the location of various epidermal stem cell niches (Figures 1 and 2), suggests that the HF *Krox20*-positive stem cells serve as the source for previously identified stem cells within the HF. To verify this, we stained for bulge stem cell markers in *Krox20-CreERT; R26-tdTomato* lineage-traced skin. As expected, the *Krox20*-lineage cells largely overlapped with K15-positive and CD34-positive bulge cells in both anagen and telogen HFs (Figure 3, A–F), consistent with previous reports of the ability of infundibular cells to regenerate the bulge structure (19). This is critical genetic evidence demonstrating that *Krox20*-positive cells are the parental/ancestral cells of *K15*-positive bulge stem cells. Quantification of CD34-positive bulge cells arisen from *Krox20*-lineage cells at different time points in *Krox20-CreERT; R26-tdTomato* mice induced at P1 is shown in Supplemental Figure 3.

### Krox20-positive cells are indispensable for hair development and regeneration.

**Figure 4:**
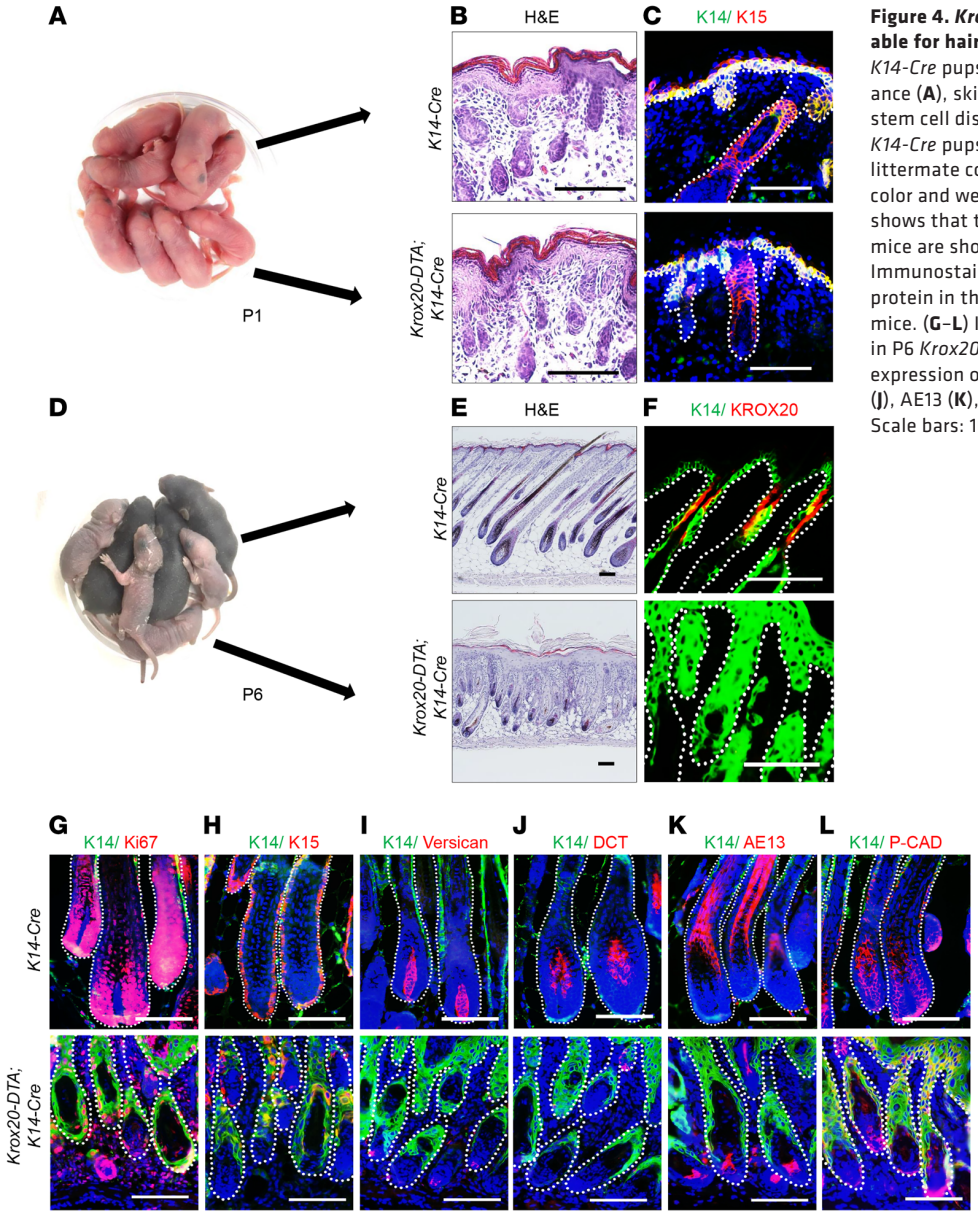
*Krox20*-positive cells are indispensable for hair development. (**A**–**C**) *Krox20-DTA; K14-Cre* pups at P1 show normal gross appearance (**A**), skin histology (**B**), and K15-positive stem cell distribution (**C**). (**D**) *Krox20-DTA; K14-Cre* pups show no difference compared with littermate controls at P1 but are lighter in skin color and weight by P6. (**E**) H&E staining at P6 shows that the HFs of *Krox20-DTA; K14-Cre* mice are shorter and random in orientation. (**F**) Immunostaining shows the absence of KROX20 protein in the skin of *Krox20-DTA; K14-Cre* mice. (**G**–**L**) Immunostaining reveals that HFs in P6 *Krox20-DTA; K14-Cre* pups have aberrant expression of Ki67 (**G**), K15 (**H**), versican (**I**), DCT (**J**), AE13 (**K**), and P-cadherin (P-CAD) (**L**). *n* = 5. Scale bars: 100 μm.

To define the functional role of epidermal *Krox20*-positive cells during development, we deleted *Krox20*-expressing cells in the skin by crossing mice harboring the *Krox20-lox-Stop-lox-DTA* (*Krox20-DTA*) knockin allele (15) with a *K14-Cre* line to generate *Krox20-DTA; K14-Cre* mice. In this model, *Krox20*-positive cells of the *K14* lineage express diphtheria toxin A (DTA) and are ablated. *K14* is expressed in the basal layer of the IFE and in the outer root sheath of the upper and middle regions of the HF (14, 20), while *K14* lineages contribute to the formation of the entire IFE and HF except for the dermal papillae (14). *Krox20-DTA; K14-Cre* mice were born phenotypically similar to control mice at birth (Figure 4A). The HF and skin of *Krox20-DTA; K14-Cre* pups at P1 were histologically normal (Figure 4B) and expressed the markers K14 and K15 in a similar pattern in comparison with the control mice (Figure 4C). These data suggest that epithelial *Krox20*-positive cells are not essential for normal embryonic skin and initial HF development. However, within 1 week of postnatal development, *Krox20-DTA; K14-Cre* pups began showing an obvious phenotype. *Krox20-DTA; K14-Cre* mice were visibly smaller by P3, and their skin pigmentation was significantly lighter by P6 (Figure 4D). As opposed to human skin, where pigmentation is derived from IFE melanocytes, skin pigmentation of postnatal mice is derived from melanin in the anagen HF, suggesting a loss or arrest of anagen HF development in the *Krox20-DTA; K14-Cre* mice. Consistent with this, histological analysis of the skin of P6 *Krox20-DTA; K14-Cre* mice showed significantly shorter HFs in comparison with their littermate controls (Figure 4E). Additionally, their HFs grew in discordant directions, while normal mouse anagen HFs grew in a similar direction (Figure 4E). The observation of miniaturized HFs was anticipated, as *Krox20*-positive cells are distributed within the upper and middle anagen HFs, and their deletion results in shorter HFs.

To validate the ablation of *Krox20*-positive cells by DTA, immunostaining was performed using the KROX20 antibody. The results of the immunostaining clearly demonstrated the complete absence of KROX20 expression, confirming that epithelial *Krox20*-positive cells were effectively eliminated in the examined samples (Figure 4F). Complete absence of *Krox20*-expressing cells, despite the partial overlap between *K14* and *Krox20*, suggests that the ablated population of *Krox20*-positive cells in *K14* lineages may give rise to the *Krox20*-expressing cells that do not express *K14*. This observation is consistent with our previous report, in which we proposed a pattern indicating that embryonic *K14*-positive cells serve as the origin for a specific subset of *Krox20*-expressing cells (14).

Normal anagen HFs are associated with highly proliferative cells in the matrix, also known as transit-amplifying cells, that are derived from bulge stem cells (21). To look for the presence of highly proliferative cells, we performed immunostaining for Ki67. Mice lacking epidermal *Krox20-*positive cells showed no matrix transit-amplifying cells (Figure 4G), further supporting the role of *Krox20*-positive cells as HF stem cells upstream of transit-amplifying cells. To explore the effect of *Krox20*-positive cell depletion on the cells and structures of the HF, we performed immunostaining for K15, a bulge stem cell marker (6). We found that loss of *Krox20*-positive cells resulted in significantly reduced *K15*-positive cells (Figure 4H), suggesting that *Krox20*-positive cells are the ancestral cells of *K15*-positive cells within the HF.

The dermal papilla is a mesenchymal structure deep in the HF that reciprocally signals with HF stem cells (22) to initiate and maintain normal HF growth state (23). To determine whether loss of epidermal *Krox20*-positive cells affects the dermal papillae, we stained for the marker versican. We found atrophied dermal papillae in *Krox20*-positive cell–deficient HFs (Figure 4I). Given that *Krox20*-positive cells were depleted only in the epidermal *K14*-positive lineages, our results provide further support for the existence of cellular crosstalk between the HF epidermis and mesenchyme. Melanocytes are another cell type associated with HF development. Melanocyte stem cells reside in the bulge (24), and their differentiation is synchronized with the hair cycle. During normal anagen, differentiated melanocytes (DCT-positive cells) were predominantly located in the HF bulb (Figure 4J). Depletion of *Krox20*-positive cells resulted in loss of melanocytes from the bulb, suggesting that *Krox20*-positive cells either give rise to melanocyte stem cells or are essential for their differentiation (Figure 4J).

Lastly, we explored the role of *Krox20*-positive cell depletion in hair shaft formation. Loss of *Krox20*-positive cells resulted in impaired hair shaft abundance and structure, as demonstrated by reduced and aberrant immunostaining for the hair shaft structural markers AE13 and P-cadherin (Figure 4, K and L). Given that HFs of *Krox20-DTA; K14-Cre* mice are normal at birth, our findings suggest that *Krox20*-positive cells differentiate to replace the embryonic hair shaft progenitors, as well as maintain normal HF structure after birth. Taken together, our data reveal a requirement for epidermal *Krox20*-positive cells in HF morphogenesis. They also indicate an essential role for *Krox20*-lineage cells as supported by the absence of markers for dermal papillae, matrix transit-amplifying cells, differentiated melanocytes, and hair shafts, which are regions devoid of *Krox20* expression.

### Krox20-positive cells repopulate the bulge and are required for hair regeneration.

**Figure 5:**
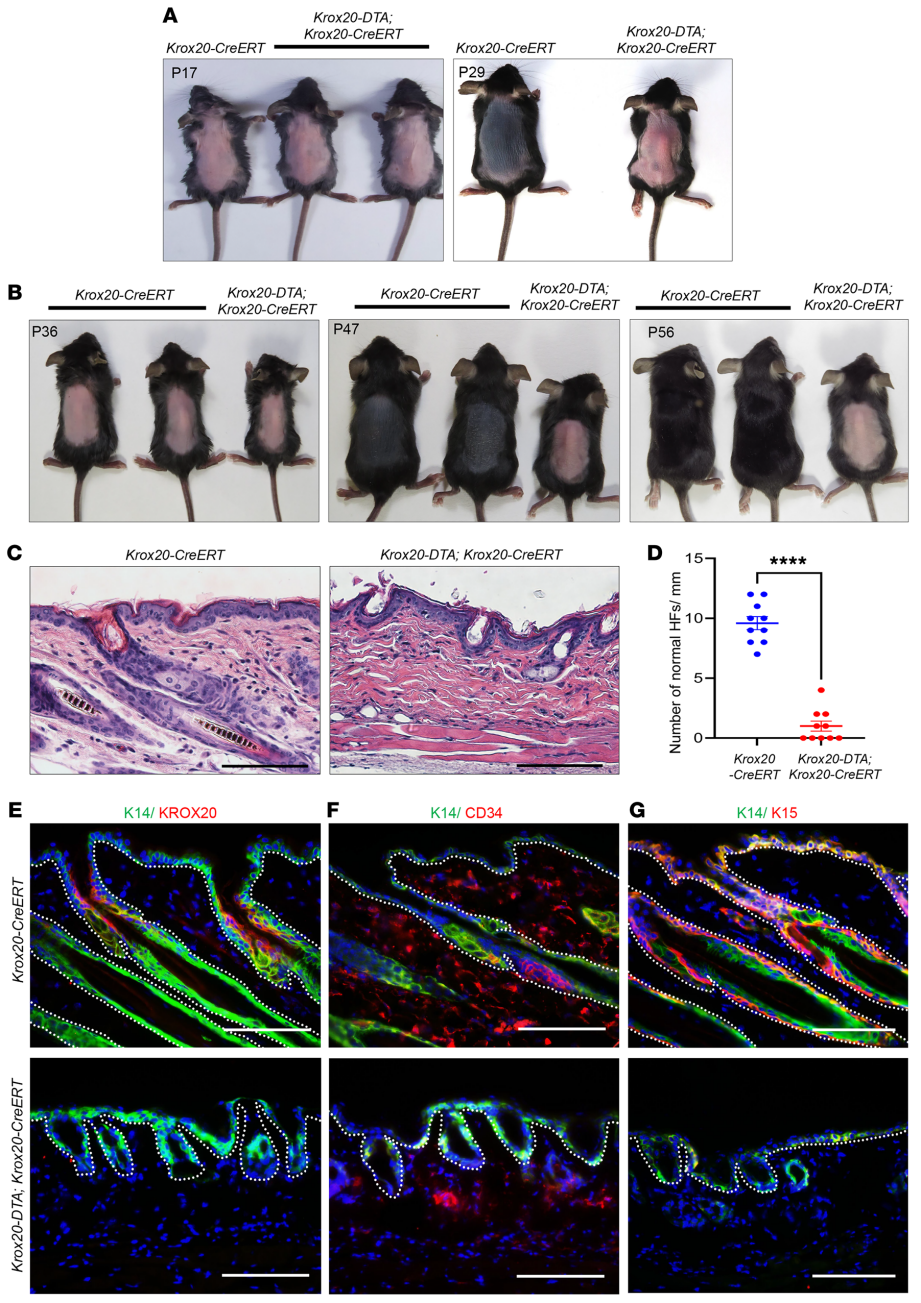
*Krox20*-positive cells are essential for hair regeneration. (**A** and **B**) Ablation of *Krox20*-positive cells via tamoxifen induction in *Krox20-DTA; Krox20-CreERT* mice during telogen I (P17) (**A**) and telogen II (P36) (**B**) hinders hair regeneration following depilation (*n* = 3). (**C**) H&E analysis of the skin in telogen II *Krox20*-positive cell–depleted mice shows hair growth arrest and HF miniaturization. (**D**) Quantification of number of normal HFs per millimeter of skin cross section of *Krox20* cell–depleted and littermate control mice (*n* = 2). (**E**–**G**) Immunofluorescence staining at P56 reveals the absence of KROX20-positive cells (**E**), and bulge CD34-positive and K15-positive cells (**F** and **G**). *n* = 3. Scale bars: 100 μm. Statistical significance was determined for **D** by unpaired 2-tailed Student’s *t* test; statistics represent mean ± SEM, ****P < 0.0001.

*Krox20-DTA; K14-Cre* mice do not survive beyond 1 week of age, most likely because of epithelial dysregulation of vital internal organs, and thus we were unable to determine the adult epidermal phenotype. To examine the effects of *Krox20*-lineage cell depletion on hair regeneration in adult mice, we used a 4-hydroxytamoxifen–inducible mouse model (*Krox20-DTA; Krox20-CreERT*) to deplete *Krox20*-expressing cells in vivo. In this model, *Krox20*-expressing cells express DTA and are ablated upon induction with 4-hydroxytamoxifen. The expression of *Krox20* is preserved in *Krox20-DTA; Krox20-CreERT* mice because of the design of the *Krox20-CreERT* line using the P2A linker (Supplemental Figure 2A). We used this model to ablate *Krox20*-expressing cells in depilated HFs at various stages of the hair cycle: Mice were depilated to cause synchronized induction of new hair generation at P17, P23, P36, P38, P55, and P104. 4-Hydroxytamoxifen dissolved in DMSO was topically administered to the depilated dorsal skin at the same time for a minimum of 3 consecutive days. Interestingly, the effect of depleting *Krox20*-expressing cells on hair regeneration varied depending on the hair cycle stage at the time of induction. The most prominent phenotype was observed when depilation and 4-hydroxytamoxifen induction were initiated during early telogen I and II, at P17 and P36, respectively (Figure 5): At both hair cycle stages, *Krox20-DTA; Krox20-CreERT* mice lost their ability to generate new hair, with the hair cycle arrested in telogen, while the HFs of littermate controls progressed to anagen (Figure 5, A and B). Histological analysis of *Krox20*-positive cell–depleted HFs at telogen II showed severely miniaturized and deformed HFs (Figure 5, C and D) that lacked KROX20 protein expression (Figure 5E), confirming the ablation of *Krox20*-positive cells. Significantly fewer normal HFs were observed per millimeter of skin cross section from *Krox20-DTA; Krox20-CreERT* mice compared with littermate controls (Figure 5D). Furthermore, immunostaining revealed complete absence of CD34-positive and K15-positive bulge stem cells (Figure 5, F and G). In addition to suggesting that *Krox20*-positive cells are the stem/progenitor cells that give rise to HF stem cells, these results also demonstrate that *Krox20*-lineage cells are essential for maintaining these HF stem cell niches during early adulthood.

In addition to telogen II, we also evaluated how hair regeneration is affected when *Krox20*-expressing cells are depleted during anagen II (P38). Interestingly, we found that *Krox20*-positive cell–depleted HFs were initially arrested at anagen II, while HFs in control mice completed the anagen phase and proceeded to telogen (Supplemental Figure 4A, middle). However, signs of hair loss were observed 2 weeks after induction, and these mice showed significant hair loss by P58 (Supplemental Figure 4A). Histological analysis using H&E (Supplemental Figure 4, B and C) and immunofluorescence staining (Supplemental Figure 4D) revealed the presence of both miniaturized and full-length telogen HFs, expressing KROX20, although at reduced levels compared with the control telogen HFs. The presence of KROX20 expression in these HFs was likely due to the incomplete ablation of *Krox20*-positive cells. Additionally, we immunostained for bulge stem cell markers CD34 and K15. While CD34 expression was lost from the HFs (Supplemental Figure 4E), K15 expression was still detected within the *Krox20*-positive cell–depleted HFs (Supplemental Figure 4F). However, it is important to note that the expression pattern of K15 in these HFs did not structurally resemble a normal bulge region, which is consistent with the absence of CD34 expression (Supplemental Figure 4, E and F). Moreover, in line with the consequences of *Krox20*-positive cell depletion during telogen II, a significant reduction was observed in the number of normal HFs per millimeter of skin cross section in the anagen II cycle of *Krox20-DTA; Krox20-CreERT* mice induced with 4-hydroxytamoxifen, compared with the control mice (Supplemental Figure 4C). Furthermore, ablation at P23, P55, or P104 showed no difference in hair regeneration capacity in the *Krox20-DTA; Krox20-CreERT* mice compared with the littermate control mice (Supplemental Figures 5 and 6).

To investigate the reasons behind the failure of depilation-induced hair regeneration when *Krox20*-positive cells were ablated at early telogen I and telogen II, but not at other tested time points, we conducted lineage tracing in *Krox20-CreERT; R26-tdTomato* mice. Tamoxifen induction was performed at P17, P23, P38, P55, and P98. Interestingly, lineage tracing during both late catagen/early telogen I (P17) and late anagen/early catagen II (P38) revealed labeling of the bulge cells by *Krox20*-lineage cells within only 4 or 3 days following tamoxifen induction, respectively (Supplemental Figure 4G and Supplemental Figure 5A). Conversely, lineage tracing at mid–telogen I (P23) did not exhibit bulge cell labeling until after telogen II (P55) (Supplemental Figure 5C). Lineage tracing at mid–telogen II (P55) demonstrated that *Krox20*-lineage cells had not yet reached the bulge even 59 days later (Supplemental Figure 6B). Furthermore, lineage tracing at P98 indicated that *Krox20*-lineage cells did not reach the bulge until 80 days later (Supplemental Figure 6D). These results suggest that *Krox20*-positive cells replenish the bulge stem cells during early telogen I and telogen II, which represent highly synchronized hair cycles.

In experiments in which mice older than 1 month were depleted of *Krox20*-expressing cells, their survival was limited to 3–4 weeks after the last dose of 4-hydroxytamoxifen. However, mice treated during telogen I exhibited a higher mortality rate (note that in Figure 5A, only 1 mouse of the 2 treated survived the 4-hydroxytamoxifen treatment to reach the age of P29) and survived no more than 10–14 days after the final administration of 4-hydroxytamoxifen. Notably, all mice displayed signs of emaciation, kyphosis, shivering, and impaired coordination 2–3 days before death. The observed phenotype was likely due to the crucial role of *Krox20* in maintaining the myelinating state of Schwann cells (25). Hence, we were unable to extend our assessment of skin phenotypes in *Krox20-DTA; Krox20-CreERT* mice beyond the time points documented in this study.

### Contribution of Krox20-lineage cells to the first formation of the bulge.

Our finding that depletion of *Krox20*-expressing cells at P17 disrupts hair regeneration during initial bulge formation (P18–P21) (Figure 5A) (26, 27) raises the question of how *Krox20*-lineage cells contribute to the first formation of the bulge. Previous studies proposed the existence of a quiescent, label-retaining cell population expressing *Sox9* in the pre-bulge region during early developmental stages. These bulge precursor cells persist or remain there to form the first adult bulge (28).

**Figure 6:**
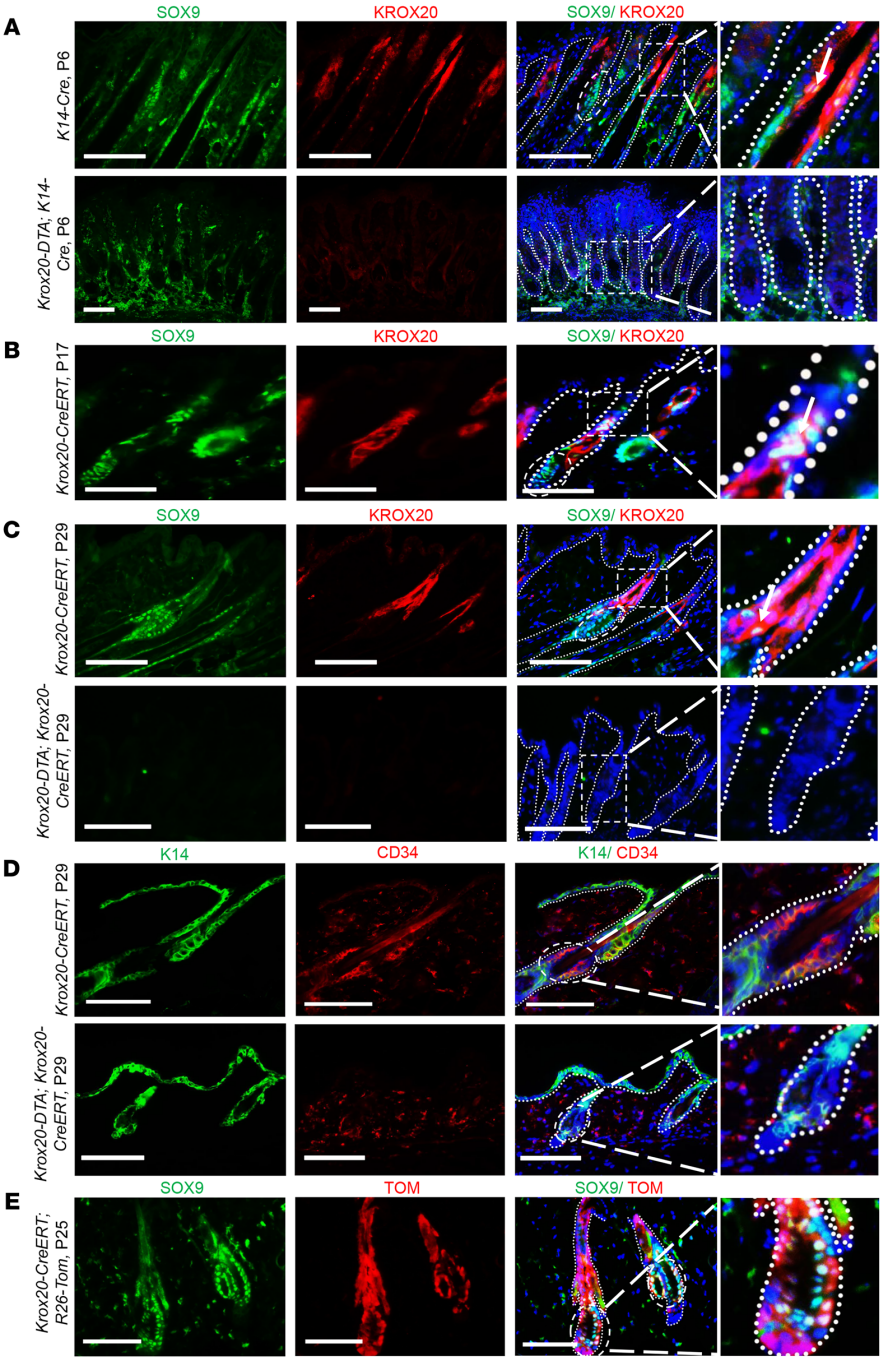
Lineages of *Krox20*- and *Sox9*-coexpressing cells in the upper HF move down to contribute to the first formation of the bulge. (**A**) Ablation of *Krox20*-positive cells in the *K14* lineage results in diminished levels of SOX9 expression within the entire HF at P6. (**B**) *Sox9*- and *Krox20*-coexpressing cells overlap in the upper and middle telogen HF at P17. (**C** and **D**) Ablation of *Krox20*-expressing cells at P17 results in the complete absence of SOX9 (**C**) and CD34 (**D**) bulge stem cell markers at P29. (**E**) *Krox20*-lineage cells encompass a population of the *Sox9*-expressing cells in the bulge. Dashed ovals represent the bulge area. Dashed rectangles represent the area coexpressing SOX9 and KROX20 in control skin, or regions devoid of KROX20 and/or SOX9 expression in KROX20-ablated skin. Higher magnifications of the areas represented by the circles or rectangles are shown in the panels at the end of each row (×200 for **A**, bottom, and **D**, bottom; and ×400 for **A**, top, **B**, **C**, **D**, top, and **E**). Arrows point to the cells coexpressing KROX20 and SOX9. *n* = 3. Scale bars: 100 μm.

Recent research also suggests that bulge stem cells derive from the periphery of the placode basal layer cells expressing *Sox9* (29), emphasizing the importance of *Sox9*-expressing cells in the first formation of bulge. We therefore investigated the spatial domains of *Sox9* and *Krox20* expression during pre-bulge formation (P6) and the onset of the first bulge (P17). At P6, representing the first anagen phase, *Sox9* expression mainly extended from the pre-bulge region to the middle of the HF (Figure 6A). A small area of *Sox9* expression could also be detected in the upper HF, where it overlapped with *Krox20* expression. It is noteworthy that *Sox9* expression was observed in the bulge region, while *Krox20* expression was detected in the innermost layer of the outer root sheath in the middle of anagen HF, surrounding the pre-bulge region without entering it (Figure 6A). Furthermore, we explored the impact of *Krox20-*expressing cell depletion on *Sox9* expression in *Krox20-DTA; K14-Cre* pups (P6). Remarkably, while the overlap of *Krox20* and *Sox9* in the upper HF was minimal, *Sox9* expression within most of the HFs was diminished in the P6 *Krox20-DTA; K14-Cre* mice (Figure 6A).

Further examination of *Krox20* and *Sox9* expression just before the onset of the initial bulge formation at P17 revealed that while *Sox9* was predominantly expressed in the bulge, and *Krox20* was excluded from it, there were overlapping domains in the upper and middle telogen HFs (Figure 6B). This temporally coincided with the time point at which the depletion of *Krox20*-expressing cells resulted in the loss of hair regeneration ability (Figure 5A). Moreover, we investigated the consequences of *Krox20* cell ablation during this time point (telogen I, P17) and observed aberrant HFs showing the complete absence of *Sox9-*expressing cells within the entire HF, including the lower HF at P29 (Figure 6C). These HFs lacked a structurally defined bulge and did not express the bulge stem cell marker CD34 (Figure 6D). Lineage tracing of *Krox20* cells at P25, just after the formation of the first bulge and induced at P17, revealed that *Krox20*-lineage cells detected in the bulge area encompassed a population of *Sox9*-expressing cells within the bulge (Figure 6E). Quantification of the observations shown in Figure 6 is presented in Supplemental Figure 7.

Given the lineage tracing results and the observation that the overlap between *Sox9-* and *Krox20*-expressing cells occurred only in the upper and middle HFs, but ablation of *Krox20-*expressing cells resulted in the absence of *Sox9* within the entire HF, we propose that the lineages of *Krox20*- and *Sox9*-coexpressing cells in the upper HF may migrate down and contribute to the initial formation of the bulge, while continuing to express *Sox9* but turning off *Krox20* expression.

### KROX20 protein is critical for epidermal tissue homeostasis.

**Figure 7:**
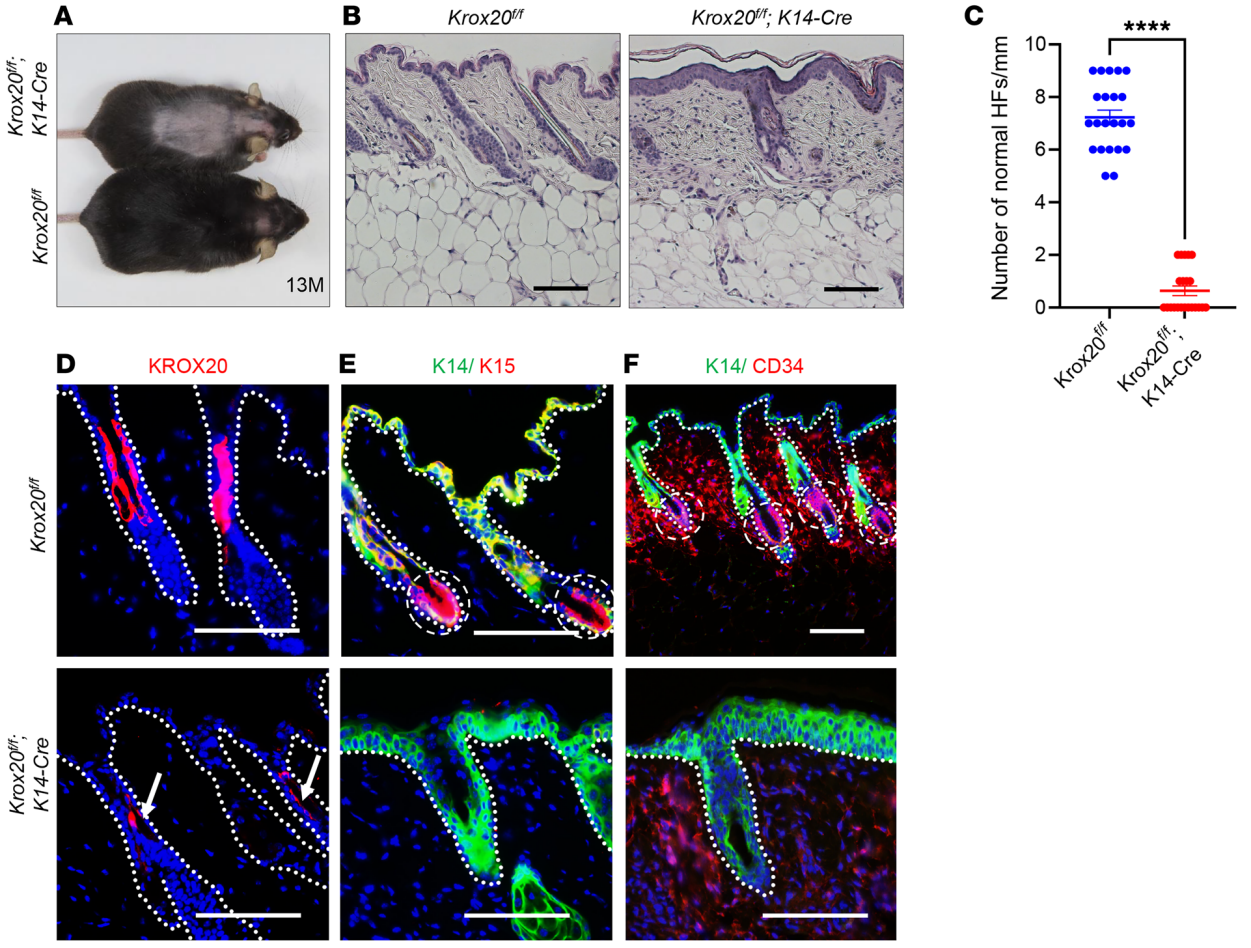
KROX20 protein is critical for epidermal tissue homeostasis. (**A**–**C**) Approximately 83% of *Krox20^fl/fl^; K14-Cre* mice show pattern hair loss during aging (**A**) (*n* = 18); in the balding skin, the majority of HFs are deformed, with some HFs arrested at anagen while others are miniaturized (**B**). (**C**) Quantification of number of normal HFs per millimeter of skin cross section of *Krox20^fl/fl^; K14-Cre* mice and their littermate controls (*n* = 7). (**D**) Immunofluorescence staining of the skin confirmed the reduced expression of epithelial KROX20 protein in *Krox20^fl/fl^; K14-Cre* mice. (**E** and **F**) The miniaturized HFs in the *Krox20^fl/fl^; K14-Cre* mice lack normal bulge structure, characterized by the absence of HF stem cell markers K15 (**E**) and CD34 (**F**). *n* = 3. Dashed circles represent the bulge area. Arrows point to the traces of KROX20 in the HFs. Scale bars: 100 μm. Statistical significance was determined for **C** by unpaired 2-tailed Student’s *t* test; statistics represent mean ± SEM, ****P < 0.0001.

Our genetic models demonstrated that while *Krox20* is expressed in the upper and middle hair follicular cells, its expression is downregulated in *Krox20*-lineage cells that differentiate and migrate to the hair shaft. This suggests that *Krox20* may be an important regulator of stem cell quiescence and a fate-specific marker of epidermal stem cells. To address the function of *Krox20* in epithelial homeostasis, we generated a *Krox20^fl/fl^; K14-Cre* mouse model. These mice displayed overall normal health and appearance during their early stages. Intriguingly, a majority of these mice exhibited hair loss specifically in the anterior dorsum around 1 year of age (Figure 7A). Although the mechanism of region-selective hair loss is unclear, histological analysis revealed a reduction in the total number of normal HFs in the alopecic area, most of which were miniaturized HFs (Figure 7, B and C). Immunostaining using the KROX20 antibody confirmed that deletion of *Krox20* led to greatly reduced KROX20 expression in the *Krox20^fl/fl^; K14-Cre* mice (Figure 7D). Immunostaining analysis showed that the miniaturized HFs in the mutant mice lacked the bulge stem cell markers K15 and CD34 (Figure 7, E and F). Additionally, an interesting finding was that apart from the absence of *K15*-positive cells in the HFs, the *Krox20*-deficient alopecic epidermis also exhibited a loss of *K15*-positive cells in the IFE (Figure 7E), phenocopying *Krox20*-positive cell depletion in vivo (Figure 4H). These findings suggest that the KROX20 protein plays a vital role in the functions of *Krox20*-positive cells in HF maintenance. Taken together, our data demonstrate that *Krox20* is critical for hair regeneration and homeostasis.

## Discussion

In this study, we report the transcription factor *Krox20* as a marker that is enriched in an adult epidermal stem cell population located in the HF. In a previous study, we conducted lineage tracing experiments using *K14-Cre; R26-YFP*, which indicated that embryonic *K14*-positive cells serve as the origin of a specific subset of *Krox20-*expressing cells (14). These *Krox20*-positive cells are predominantly located within the infundibulum, extending downward to the middle HF. Notably, they partially overlap with the *Sca1*-positive and *Lrig1*-positive cells, constituting a heterogeneous population of stem cells.

The phenotypic and histological analysis of the pups of *Krox20-DTA; K14-Cre* mice suggested that epithelial *Krox20*-positive cells were not essential for normal embryonic initial HF development. To assess the contribution of *Krox20*-positive cells in the generation and replenishment of bulge cells throughout life, we performed lineage tracing using inducible *Krox20-CreERT; R26-tdTomato* mice. Perinatal induction demonstrated that the HF *Krox20*-lineage cells contribute to the first formation of the bulge and its continuous maintenance. Further experiments suggested that the populations of cells expressing both *Krox20* and *Sox9* in the upper HF may move downward during the onset of the first telogen and play a role in the initial development of the bulge (Figure 6). On the other hand, when induction was performed in adulthood after HF morphogenesis was complete (Supplemental Figures 5 and 6), *Krox20*-positive cells were still observed to contribute to the bulge. The results of lineage tracing initiated perinatally and during adulthood suggest that *Krox20*-positive cells are not only essential for normal HF morphogenesis, but periodically generate and replenish HF bulge stem cells throughout life. Our data further support this scenario by demonstrating that tamoxifen-induced depletion of *Krox20*-positive cells in *Krox20-DTA; Krox20-CreERT* mice during the early telogen I, late anagen II, or early telogen II stage disrupts bulge formation, as evidenced by immunofluorescence staining with bulge stem cell markers. Therefore, we conclude that *Krox20*-positive cells contribute to the formation of adult HF and hair shaft. One explanation for why *Krox20*-lineage cells migrate downward to replenish the bulge at the onset of telogen I and II may be the rapid and synchronous nature of first and second hair cycles. However, after telogen II, since hair cycles are no longer synchronous, identifying the time point to ablate *Krox20*-expressing cells when most HFs complete the anagen stage and enter catagen/telogen becomes challenging. An alternative explanation might be that the contribution of *Krox20*-lineage cells to the bulge may change with time, and after contributing to the first and second bulge formation, the bulge stem cell reservoir may not need replenishment from the *Krox20*-lineages. We conclude that *Krox20*-positive cells contribute to the bulge formation. This is supported by several key pieces of evidence. First, costaining of *Krox20*-lineage-traced HFs demonstrated that *Cd34*-positive/*K15*-positive bulge stem cells are contained within the *Krox20* lineage. Second, ablation of *Krox20*-positive cells results in loss of the bulge stem cell population and failure of hair regeneration at certain hair cycle stages. Third, ablation of epithelial *Krox20*-positive cells or loss of *Krox20* expression in *K14*-positive cells also results in the depletion of bulge stem cells, leading to hair growth arrest. These data indicate that *Krox20*-positive cells are the ancestral cells of the bulge stem cells.

Findings from previous studies provide evidence that aligns with our claim, demonstrating that when bulge cells were subjected to laser ablation, epithelial cells in the infundibulum exhibited proliferative behavior, and some cells swiftly migrated into the bulge. These discoveries suggest the intriguing possibility that neighboring epithelial cells located in the upper HF might play a role in the regeneration and recovery of the bulge (19).

Our lineage tracing experiments have also provided additional evidence regarding the participation of *Krox20*-lineage cells in hair shaft generation, demonstrating their involvement in the formation of the first hair shaft as early as P12. It is widely accepted that bulge stem cells are the primary HF adult stem cells responsible for hair shaft formation. However, contribution of *Krox20*-lineage cells to hair shaft formation happens before the first formation of the bulge at approximately P18–P21 (26, 27). This observation suggests that even though the anatomical structure of the bulge niche is not established until late anagen I, the specification of stem cells within this niche occurs during the early stages of HF morphogenesis, as suggested by previous studies (28, 29).

Our lineage tracing analyses initiated at different yet overlapping time points, as depicted in Figure 2A (initiated at P1) and Supplemental Figure 6, B and D (initiated at P55 and P98, respectively), provide valuable insights into the understanding of our lineage tracing data. Upon tamoxifen induction between P98 and P100, *Krox20*-lineage cells were predominantly observed in the upper HF at 1 day after induction (P99), with virtually no presence in the bulge (Supplemental Figure 6D). In contrast, in mice of a similar age (P100) induced at P1, *Krox20*-lineage cells had already extensively labeled the bulge area (Figure 2A). Similarly, when induction was conducted at P55, *Krox20*-lineage cells were not detected in the bulge area even 59 days after induction (Supplemental Figure 6B). However, mice induced at P1 exhibited labeling of the bulge region at comparable ages (Figure 2A). These observations significantly reduce the likelihood that the *tdTomato* signal in the bulge area is due to low-level expression of *Krox20*. However, the available single-cell RNA sequencing data sets suggest a low expression level of *Krox20* in the bulge (30) (https://kasperlab.org/tools) using bulge stem cell markers. Based on the same data sets and our immunofluorescence staining (Figure 1, D and E, and Figure 6, A–D), all known bulge stem cell markers, such as *Sox9*, *Nfatc1*, *K15*, and *Cd34*, are expressed in the upper HF and may overlap with *Krox20* outside of the bulge region. Therefore, these markers may not be accurately demonstrating the expression of *Krox20* within the bulge, but rather reflecting overlap of these “bulge markers” with *Krox20* outside of the bulge. Single-cell RNA sequencing data lack the spatial resolution required to distinguish between the bulge and non-bulge regions, potentially leading to misinterpretation of a gene expression domain. Given that single-cell RNA sequencing detects mRNA rather than protein, we consider immunofluorescence a more reliable method for assessing *Krox20* expression in the bulge.

Nonetheless, the possibility of low *Krox20* expression in the bulge, below the detection threshold of our immunofluorescence staining with KROX20 or GFP antibody (Figure 1) or live GFP expression in *Krox20-GFP* (Supplemental Figure 1C), cannot be totally excluded. It is also possible that *Krox20*-lineage cells migrating from the upper HF to the bulge region are still expressing *Krox20* at a very low level. To check for *Cre* expression, we stained skin sections from *Krox20-CreERT; R26-tdTomato* mice induced at P23, at different times after tamoxifen induction, with Cre antibody. The patterns of Cre staining recapitulate those of KROX20 (Supplemental Figure 8, A–D), and no Cre staining was detected in the bulge region of the HFs (Supplemental Figure 8E). Additionally, *tdTomato* never labeled the bulge region immediately after tamoxifen induction (Figure 1E, Supplemental Figure 5, A and C, and Supplemental Figure 6, B and D). This suggests that even if the bulge cells express a low basal level of *Cre*, they are not *tdTomato*-labeled with tamoxifen induction, and their contribution to the bulge is likely not traced during the lineage tracing. This approach is used in lineage tracing, where induction is adjusted so that only highly expressing cells are labeled and tracked for their fate (31, 32).

Additionally, our lineage tracing experiments initiated at P24, P55, and P99 reveal bulge labeling long after tamoxifen induction. For instance, after induction at P24, bulge labeling takes nearly a month (Supplemental Figure 5C); after induction at P99, it takes 78 days; and after induction at P55, the bulge is not labeled even after 59 days (Supplemental Figure 6, B and D). While there is a slight possibility that cells expressing *Cre* at basal levels require more time for Cre to reach the threshold necessary for recombination, this is highly unlikely to happen in our inducible *Krox20-CreERT; R26-tdTomato* mice, where *tdTomato* labeling can only occur when CreERT translocates to the nucleus in the presence of tamoxifen. Given that the potential for leakiness was ruled out (Supplemental Figure 2), detection of *tdTomato* signal in the bulge region in the absence of tamoxifen suggests that this labeling is not due to late recombination resulting from low *Cre* expression long after induction. Instead, it most likely reflects the migration of lineage-labeled cells to the bulge area.

Our data also suggest that *Krox20*-lineage cells might give rise to the IFE in adult mice. However, as the concept of a stem cell population giving rise to both the bulge and IFE represents a paradigm shift from what is established in the literature, we aim to further investigate observed expression of *tdTomato* within the IFE. Those studies would be intended to conclusively demonstrate whether *Krox20*-lineage cells are contributing to the IFE. Following a recent study showing the presence of *Krox20* in human HFs (33) at locations similar to those observed in the mouse HF, our future goal is to explore the potential of reviving *Krox20*-positive cells in alopecic scalp. We aim to investigate whether this reactivation could be a practical and effective therapy for regrowing human hair.

## Methods

### Sex as a biological variable.

Our study examined male and female animals, and similar findings are reported for both sexes.

### Mice.

*K14-Cre* (34) mice were purchased from The Jackson Laboratory. The *Krox20-flox* (35) mouse line was provided by Piotr Topilko (Institut Mondor de Recherche Biomédicale, Créteil, France). *Krox20-flox-GFP-flox-DTA* (15), a gift from Patrick Charnay (Institut de Biologie de l’École Normale Supérieure, Paris, France) and Piotr Topilko, is a knockin allele, with GFP serving as a reporter for the *Krox20* promoter in the absence of Cre whereas in the presence of Cre, GFP is floxed out and DTA is turned on to ablate the cells. In this study, we used *Krox20-GFP* to represent this allele in the *Krox20* live expression experiments, and *Krox20-DTA* was used when Cre was introduced to ablate *Krox20*-positive cells. *Krox20-CreERT* mice were generated by the Children’s Research Institute Transgenic Core at University of Texas Southwestern Medical Center. *R26-tdTomato* mice (strain 007914) were from The Jackson Laboratory.

### Mouse hair depilation.

Mice were first anesthetized by intraperitoneal injection of 30 mg/mL ketamine and 4 mg/mL xylazine cocktail (100 μL for a 25 g mouse). The dorsal skin hair was then shaved using an electric trimmer, and depilatory cream (Nair) was applied to the trimmed area. Depilatory cream was then washed off with water.

### 4-Hydroxytamoxifen and tamoxifen induction.

4-Hydroxytamoxifen (Sigma-Aldrich) was dissolved in DMSO at 5 mg/mL. For induction of *Krox20*-lineage cell depletion in *Krox20-DTA; Krox20-CreERT* mice, 10–20 mL of 4-hydroxytamoxifen solution was applied to the depilated back skin once a day for a minimum of 3 consecutive days. For lineage tracing with *Krox20-CreERT; R26-tdTomato* mice, adult mice were given tamoxifen (1–2 mg) by gavage once a day for 3 consecutive days. For perinatal induction, 1- to 3-day-old pups received a single injection of 4-hydroxytamoxifen (40 μg) at the tail junction.

### Histology analysis.

Mouse dorsal skin was harvested and fixed in 10% formalin overnight. Samples were subjected to paraffin embedding and then sectioned at 5–8 μm thickness. H&E staining was performed following the manufacturer’s protocol (StatLab).

### Immunostaining.

For immunofluorescence staining, frozen sections or paraffin sections after deparaffinization, rehydration, and antigen retrieval were used. The primary antibodies used in this study were: AE13 (MA1-5765, Thermo Fisher Scientific); CD34 (clone RAM34, 553731, BD Pharmingen); DCT (PEP8h, gift from Vincent Hearing, NIH, Bethesda, Maryland, USA) (36); GFP (1020, Aves); K14 (NBP234675B, biotin-labeled, Novus Biologicals); K15 (ab52816, Abcam); Ki67 (15580, Abcam); KROX20 (27814, Invitrogen); LRIG1 (AF3688, R&D Systems); P-cadherin (AF761, R&D Systems); RFP/tdTomato (600-401-379S, Rockland); SCA1 (ab124688, Abcam); SOX9 (14-9765-82, Invitrogen); Cre (NB100-56133, Novus Biologicals), and versican (AB1033, Sigma-Aldrich). For immunofluorescence staining, the primary antibodies were detected by secondary antibodies or streptavidin conjugated with Cy3 or Alexa Fluor 488 (Jackson ImmunoResearch), and nuclei were counterstained with DAPI (Vector Laboratories).

### Microscopy.

Fluorescence microscopy images were taken on an Olympus fluorescence microscope (Model IX73) with software cellSens Standard (version 1.8). Images were processed by Adobe Photoshop CS6 (version 13.0.1 × 32); procedures were limited in overall brightness/contrast adjustment and multicolor channel overlay. Statistical analysis was performed by GraphPad Prism 8.

### Statistics.

Statistical analyses were performed using 2-way ANOVA or unpaired 2-tailed Student’s *t* test, as specified in figure legends (Prism 8, GraphPad). Data represent mean ± SEM. *P* values less than 0.05 were considered statistically significant. Significant differences are noted by asterisks (*P < 0.05, **P < 0.01, ***P < 0.001, ****P < 0.0001).

### Study approval.

Mouse care and experiments were approved by the Institutional Animal Care and Use Committee at University of Texas Southwestern Medical Center.

### Data availability.

Values for all data points in graphs are reported in the Supporting Data Values file.

## Author contributions

LQL conceptualized the study, established methodology, analyzed data, reviewed and edited the manuscript, and performed project management. EG, ET, TS, YW, JR, YZ, CPL, and LQL performed investigations and data acquisition. EG, ET, SL, RMM, CPL, and LQL wrote and edited the original draft. EG, ET, SL, ZC, CPL, and LQL performed visualization and data presentation. LQL performed supervision and funding acquisition.

## Supplementary Material

Supplemental data

Supporting data values

## Version 1 10/03/2024

In-Press Preview

## Version 2 12/02/2024

Electronic publication
