# Supplementary material for: Transcription factor KROX20 marks epithelial stem cell ancestors for hair follicle formation
Source: J Clin Invest. 2024 Oct 3;134(23):e180160. doi: 10.1172/JCI180160 (PMC11601947; doi:10.1172/JCI180160)
Supplement: Supplemental data [file jci-134-180160-s112.pdf]

Supplemental Figure 1

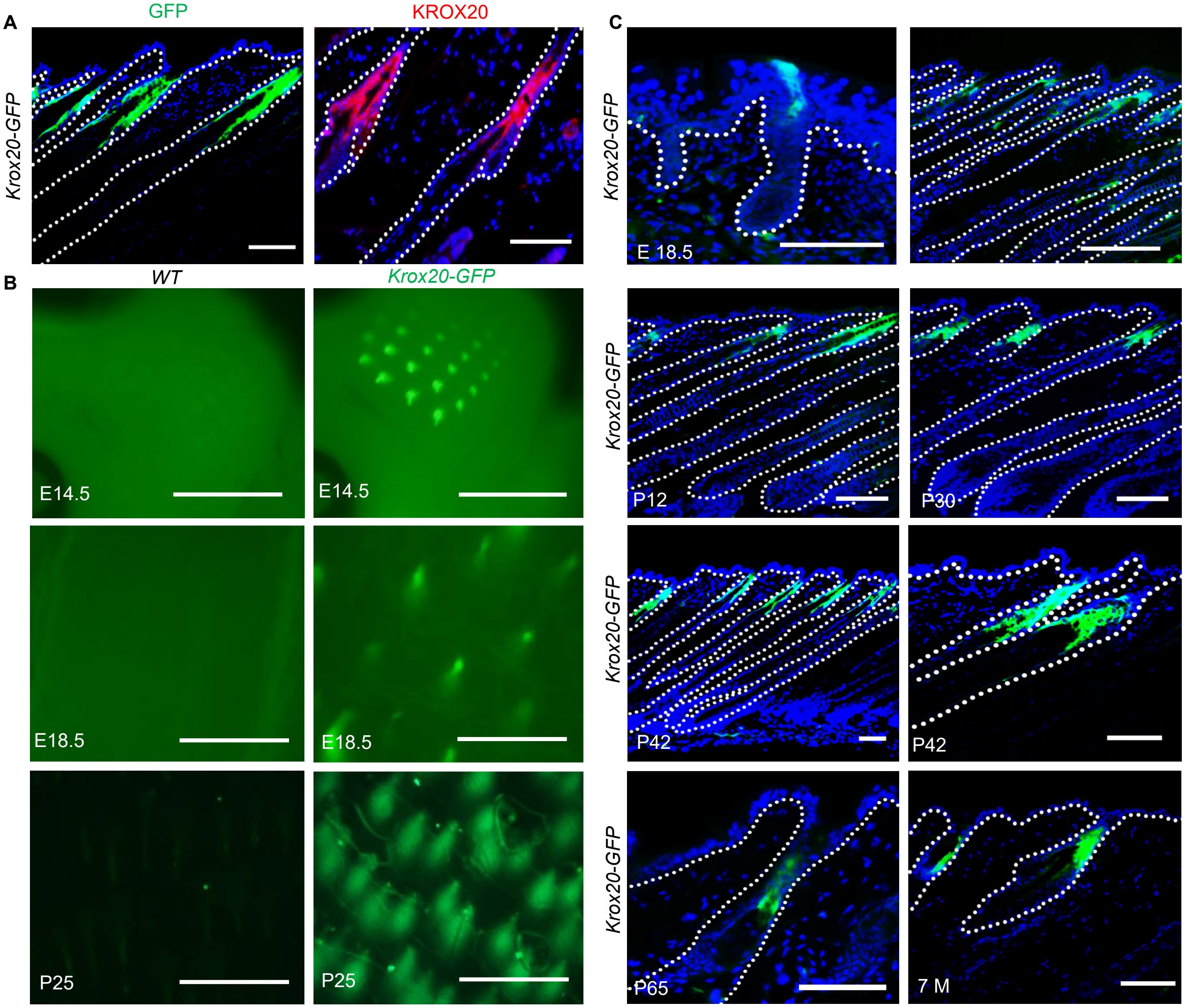

Supplemental Figure 1. KROX20 marks a niche in the upper and middle HF. (**A**) KROX20 antibody staining is restricted to the upper and middle HF similar to the live *Krox20-GFP* expression. (**B**) Whole mount imaging of *Krox20-GFP* embryos shows *Krox20* begins to express in the whisker HFs starting at E14.5 (**C**) Live expression of GFP in the dorsal skin of *Krox20-GFP* mice reveals consistent expression in postnatal HFs regardless of age. $n \geq 6$. Scale bar, 100 µm.

Supplemental Figure 2

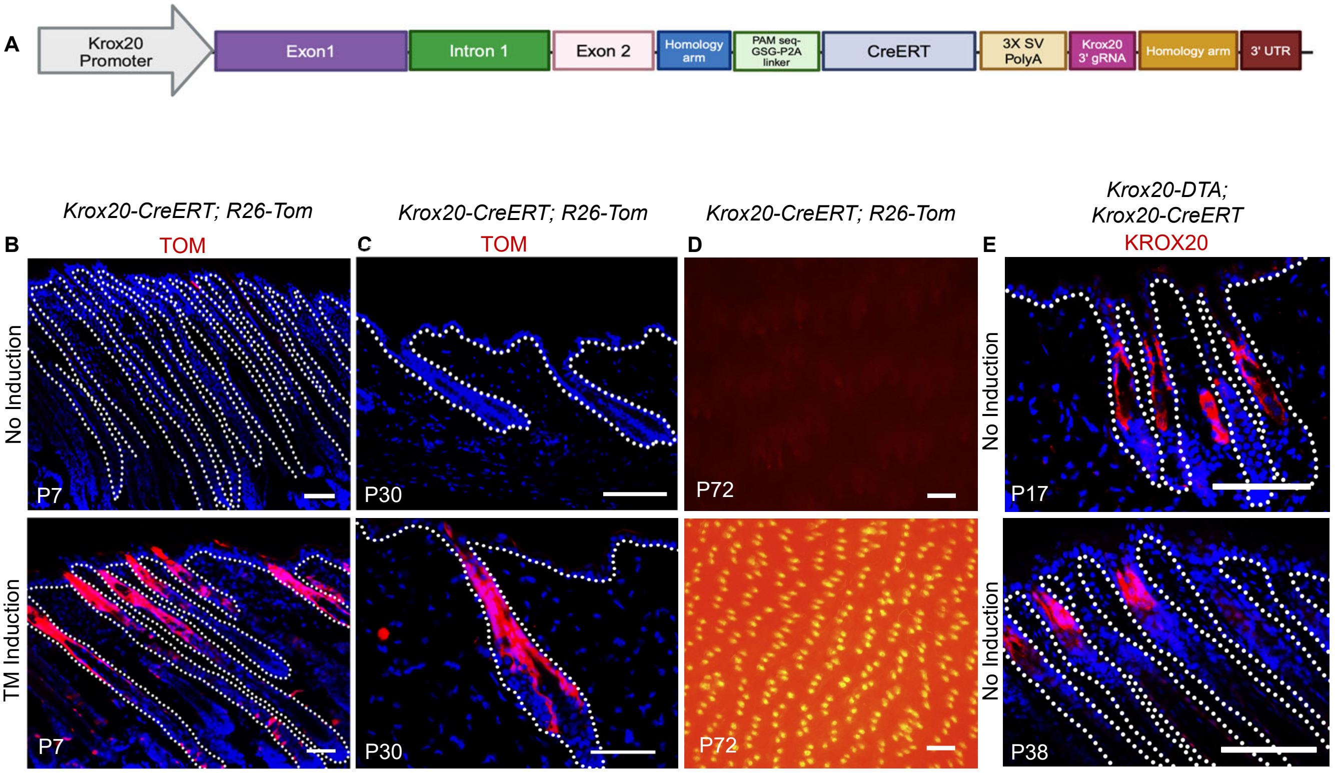

Supplemental Figure 2. Generation of an inducible *Krox20-CreERT* mouse line. (**A**) *Krox2*0 locus targeting strategy. (**B**-**E**) *Krox20-CreERT; R26-tdTomato* mice induced with tamoxifen (TM Induction) at P7, P30, or P72 showed *tdTomato* expression, while uninduced mice (No Induction) showed no *tdTomato* signal (TOM = tdTomato). (**E**) In the absence of TM induction, KROX20 is detected in *Krox20-DTA; Krox20-CreERT* mice at P17 and P38 using KROX20 antibody, demonstrating the preservation of *Krox20* expression in these mice. n = 3. TM = tamoxifen. Scale bar, 100 μm.

Supplemental Figure 3

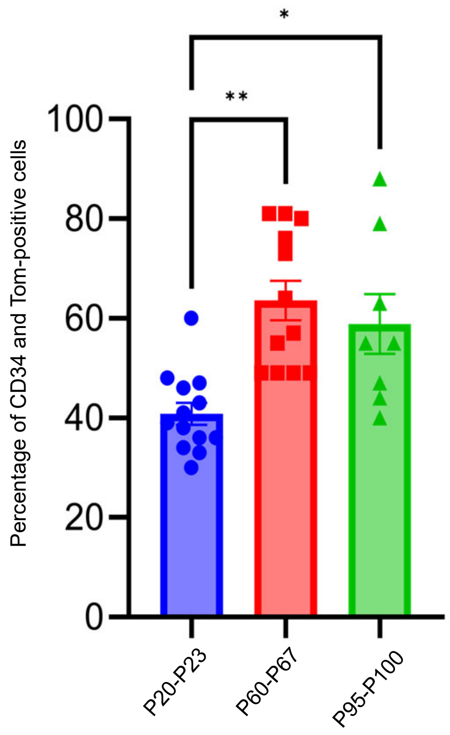

Supplemental Figure 3. *Krox20*-lineage cells overlap with CD34-positive cells in the bulge region. Quantification of CD34-positive bulge cells arisen from *Krox20*-lineage cells at different time points in *Krox20-CreERT; R26-tdTomato* mice induced at P1, as shown in Figure 3C-F. Statistical significance was determined by two-way ANOVA; statistics represent mean +SEM, $^{*}P < 0.05$, $^{**}P < 0.01$.

Supplemental Figure 4

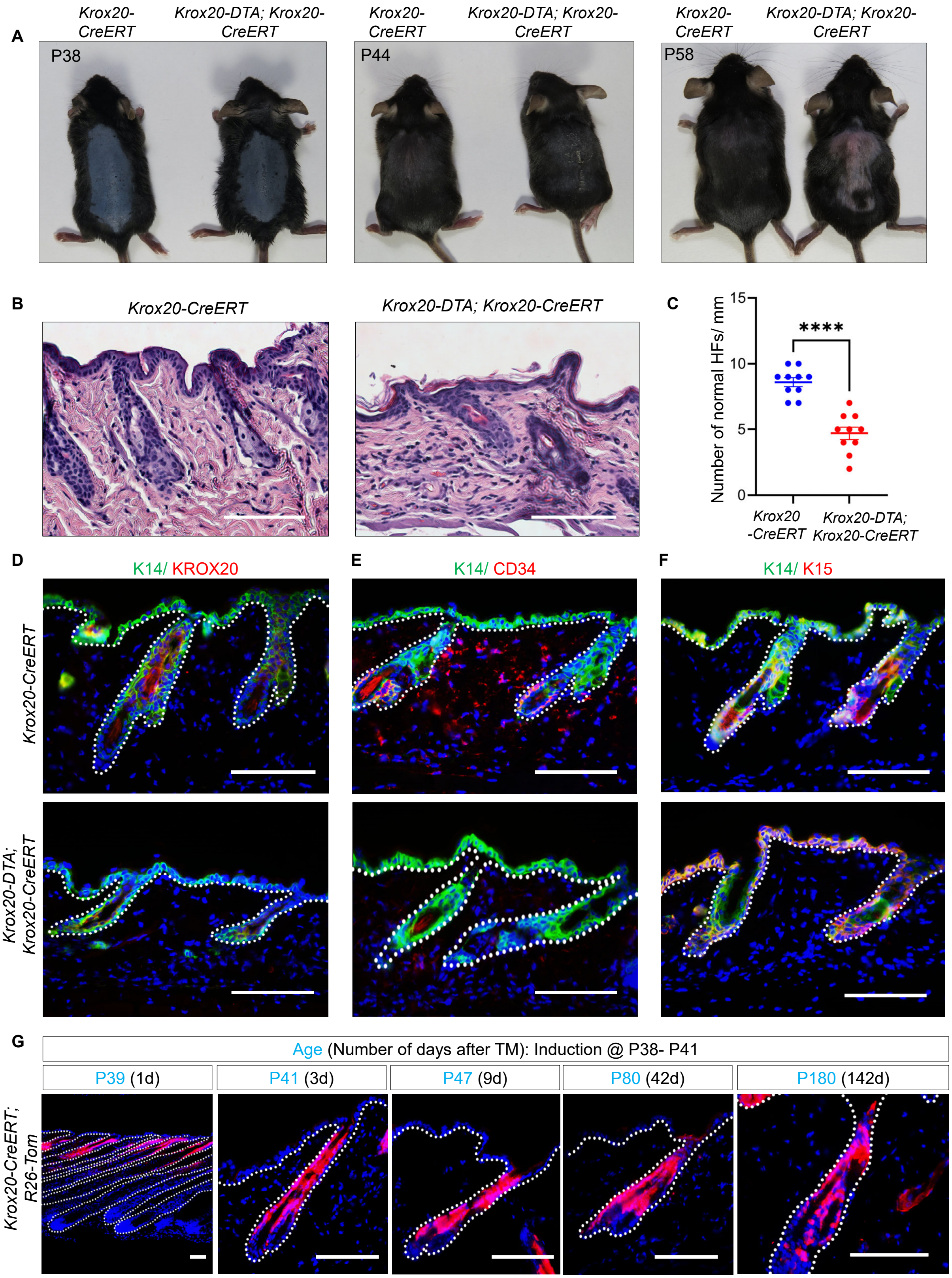

Supplemental Figure 4. *Krox20*-expressing cells are essential for hair regeneration. (**A**) Ablation of *Krox20*-positive cells via 4-hydroxytamoxifen induction in *Krox20-DTA; Krox20-CreERT* mice during Anagen II (P38) causes spontaneous hair loss and aberrant hair regeneration. (**B**) H&E analysis of the skin at P58 in these mice shows hair growth arrest and HF miniaturization. (**C**) Quantification of number of normal HFs per mm of skin cross section of *Krox20*-depleted and littermate control mice (n = 2). (**D-F**) Immunofluorescence staining at P58 reveals a reduction in *Krox20*-positive cells (**D**) and abnormal bulge structure, characterized by the absence of CD34-positive bulge stem cells (**E**). However, the expression of epidermal stem cell marker K15 (**F**) is still detected in the HF structure. (**G**) Lineage tracing using the inducible *Krox20-CreERT; R26-TdTomato* induced during the same time (P38) shows that *Krox20*-lineage cells are restricted to the upper and middle portions of the HF after 1 day of tamoxifen induction. However, by 3-days post induction, these lineage cells differentiated along the HF to the bulge and by 80-days post induction, they progressively populate the majority of the bulge. n = 3. Scale bar, 100 μm. Statistical significance for (**C**) was determined by unpaired two-tailed Student's t-test; statistics represent mean +SEM, ****$P < 0.0001$.

Supplemental Figure 5

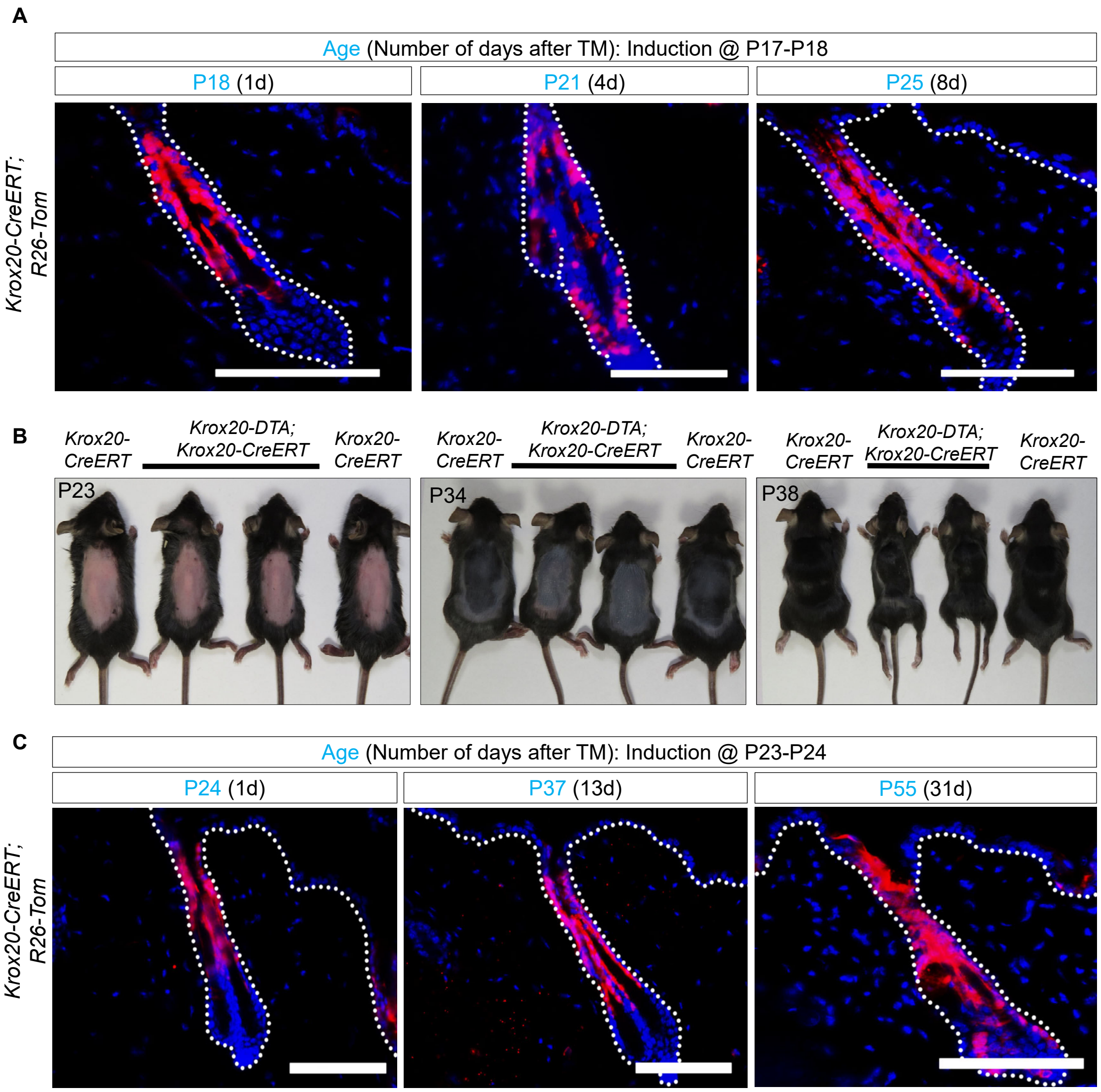

Supplemental Figure 5. The effect of depletion of *Krox20*-expressing cells on hair regeneration capacity correlates with the timing of *Krox20*-lineage cells giving rise to the bulge. (**A**) Lineage tracing analysis at P17 (early telogen I) indicates that *Krox20*-lineage cells reach the bulge by 4 days post induction. (**B**) Ablation of *Krox20*-expressing cells at P23 (mid telogen I) does not affect hair regeneration capacity. (**C**) Lineage tracing analysis at P23 indicates that *Krox20*-lineage cells reach the bulge by mid telogen II (P55). n = 3. Scale bar, 100 μm.

Supplemental Figure 6

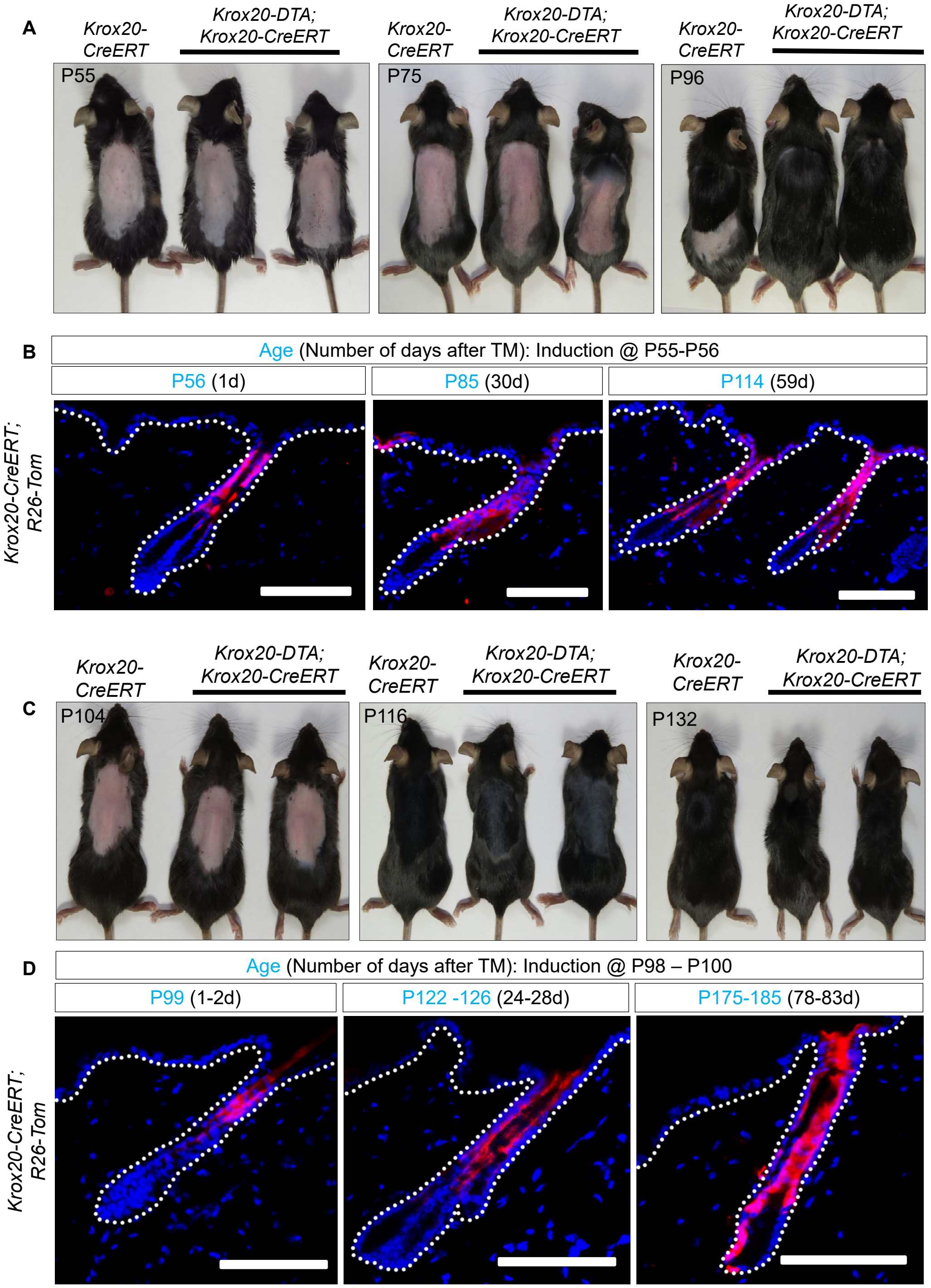

Supplemental Figure 6. The impact of ablating *Krox20*-expressing cells on hair regeneration capacity varies with hair growth cycle/phase of mice at time of 4-hydroxytamoxifen induction. (**A**) Ablation of *Krox20*-expressing cells at P55 (telogen II) does not affect hair regeneration capacity. (**B**) *Krox20*-lineage cells do not reach the bulge during the time period of the analysis in (**A**). (**C**) Ablation of *Krox20*-expressing cells at P104 does not affect hair regeneration capacity of *Krox20-DTA; Krox20-CreERT* mice. (**D**) Lineage tracing analysis at P98 shows that *Krox20*-lineage cells do not reach the bulge until 80 days after the tamoxifen induction and therefore, are not able to affect hair regeneration. n = 3. Scale bar, 100 μm.

Supplemental Figure 7

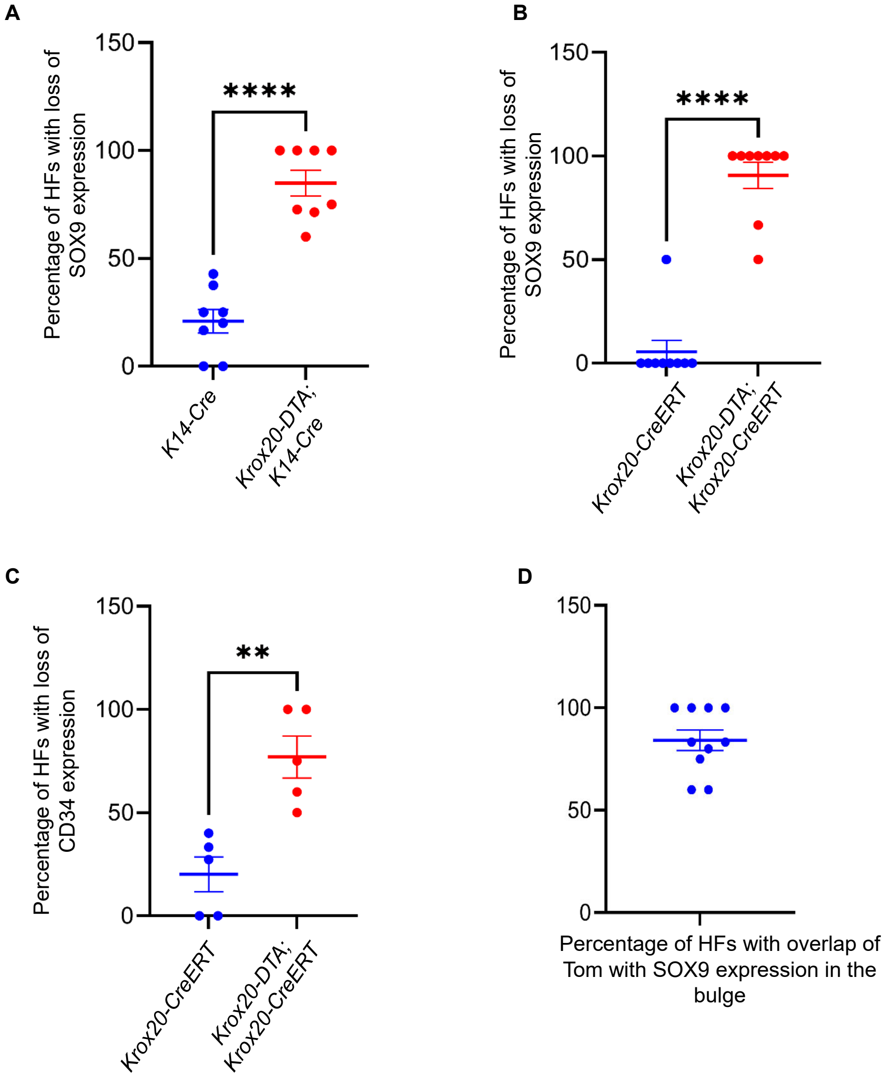

Supplemental Figure 7. Quantification of the observations shown in Figure 6. (**A**) Quantification of HFs showing loss of SOX9 expression in *Krox20-DTA; K14-Cre* mice at P6. (**B**) Quantification of HFs showing loss of SOX9 expression in *Krox20-DTA; Krox20-CreERT* mice at P29. (**C**) Quantification of HFs showing loss of CD34 expression in *Krox20-DTA; Krox20-CreERT* mice at P29. (**D**) Quantification of HFs showing overlap of *Krox20*-lineage cells (*tdTomato* positive), with SOX9 expression in the HF bulge in *Krox20-CreERT; R26-tdTomato* mice at P25. n = 3. Statistical significance was determined by unpaired two-tailed Student's t-test; statistics represent mean +SEM, $^{**}P < 0.01$, $^{****}P < 0.0001$.

Supplemental Figure 8

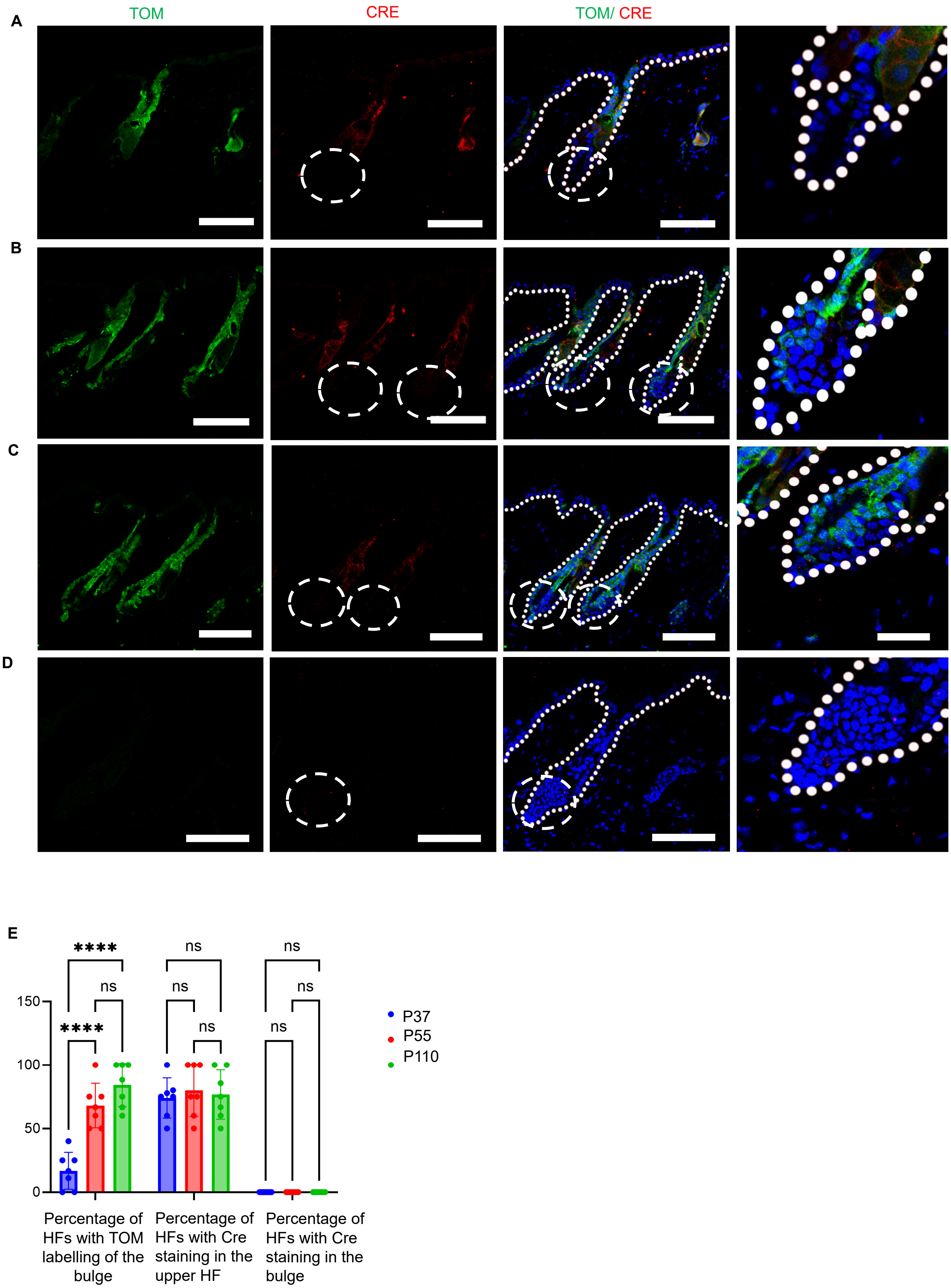

Supplemental Figure 8. Comparative analysis of *Cre* expression in telogen HFs in relation to *Krox20*-lineage cells in *Krox20-CreERT; R26-tdTomato.* (**A-C**) Skin analysis of *Krox20-CreERT; R26-tdTomato* mice induced with TM at P23 and analyzed at P37 (A), P55 (**B**) and P110 (**C**) showed that while *Krox20*-lineage cells are detected in bulge at (**B**) and (**C**), *Cre* expression is restricted to the upper HF and sebaceous glands. (**D**) *Cre-/-* skin sample shows no CRE staining. (**E**) Quantification of HFs showing labelling of the bulge and Cre expression in the upper and bulge region of HFs at time points shown in A-C. Dashed line circles represent the bulge area. TM = tamoxifen. TOM= tdTomato. n=3. Scale bar, 100 μm. Statistical significance for (**E**) was determined by two-way ANOVA; statistics represent mean +SEM, ****$P < 0.0001$.
